# ApoA-I deficiency increases cortical amyloid deposition, cerebral amyloid angiopathy, cortical and hippocampal astrogliosis, and amyloid-associated astrocyte reactivity in APP/PS1 mice

**DOI:** 10.1186/s13195-019-0497-9

**Published:** 2019-05-13

**Authors:** Emily B. Button, Guilaine K. Boyce, Anna Wilkinson, Sophie Stukas, Arooj Hayat, Jianjia Fan, Brennan J. Wadsworth, Jerome Robert, Kris M. Martens, Cheryl L. Wellington

**Affiliations:** 1 Department of Pathology and Laboratory Medicine, Djavad Mowafaghian Centre for Brain Health, University of British Columbia, 2215 Wesbrook Mall, Vancouver, British Columbia V6T 1Z3 Canada; 2 Pathology and Laboratory Medicine, Faculty of Medicine, University of British Columbia, Vancouver, BC V6T 2B5 Canada; 3 Department of Surgery, Providence Health Care Research Institute, Vancouver, BC V6Z 1Y6 Canada; 4 Department of Psychology, West Virginia University, Morgantown, WV 26506 USA

**Keywords:** Alzheimer’s disease, High-density lipoproteins, Apolipoprotein A-I (apoA-I), Amyloid beta, Cerebral amyloid angiopathy, Neuroinflammation, Astrogliosis, Cerebrovasculature

## Abstract

**Background:**

Alzheimer’s disease (AD) is defined by amyloid beta (Aβ) plaques and neurofibrillary tangles and characterized by neurodegeneration and memory loss. The majority of AD patients also have Aβ deposition in cerebral vessels known as cerebral amyloid angiopathy (CAA), microhemorrhages, and vascular co-morbidities, suggesting that cerebrovascular dysfunction contributes to AD etiology. Promoting cerebrovascular resilience may therefore be a promising therapeutic or preventative strategy for AD. Plasma high-density lipoproteins (HDL) have several vasoprotective functions and are associated with reduced AD risk in some epidemiological studies and with reduced Aβ deposition and Aβ-induced inflammation in 3D engineered human cerebral vessels. In mice, deficiency of apoA-I, the primary protein component of HDL, increases CAA and cognitive dysfunction, whereas overexpression of apoA-I from its native promoter in liver and intestine has the opposite effect and lessens neuroinflammation. Similarly, acute peripheral administration of HDL reduces soluble Aβ pools in the brain and some studies have observed reduced CAA as well. Here, we expand upon the known effects of plasma HDL in mouse models and in vitro 3D artery models to investigate the interaction of amyloid, astrocytes, and HDL on the cerebrovasculature in APP/PS1 mice*.*

**Methods:**

APP/PS1 mice deficient or hemizygous for *Apoa1* were aged to 12 months. Plasma lipids, amyloid plaque deposition, Aβ protein levels, protein and mRNA markers of neuroinflammation, and astrogliosis were assessed using ELISA, qRT-PCR, and immunofluorescence. Contextual and cued fear conditioning were used to assess behavior.

**Results:**

In APP/PS1 mice, complete apoA-I deficiency increased total and vascular Aβ deposition in the cortex but not the hippocampus compared to APP/PS1 littermate controls hemizygous for apoA-I. Markers of both general and vascular neuroinflammation, including *Il1b* mRNA, ICAM-1 protein, PDGFRβ protein, and GFAP protein, were elevated in apoA-I-deficient APP/PS1 mice. Additionally, apoA-I-deficient APP/PS1 mice had elevated levels of vascular-associated ICAM-1 in the cortex and hippocampus and vascular-associated GFAP in the cortex. A striking observation was that astrocytes associated with cerebral vessels laden with Aβ or associated with Aβ plaques showed increased reactivity in APP/PS1 mice lacking apoA-I. No behavioral changes were observed.

**Conclusions:**

ApoA-I-containing HDL can reduce amyloid pathology and astrocyte reactivity to parenchymal and vascular amyloid in APP/PS1 mice.

**Electronic supplementary material:**

The online version of this article (10.1186/s13195-019-0497-9) contains supplementary material, which is available to authorized users.

## Background

Alzheimer’s disease (AD) is a neurodegenerative disease affecting 50 million people worldwide [1] with no disease-modifying therapy [2]. Amyloid beta (Aβ) plaques and neurofibrillary tangles are the neuropathological hallmarks of AD, and Aβ is the most common target in ongoing clinical trials [2]. The majority of AD patients have co-morbid vascular diseases and cerebrovascular pathologies including microinfarcts, cerebral atherosclerosis, and arteriolosclerosis [3, 4]. Most also have Aβ deposition in cerebral arteries, known as cerebral amyloid angiopathy (CAA) [5]. Promoting cerebrovascular resilience may therefore be an attractive approach towards treating or preventing AD.

Plasma high-density lipoproteins (HDL) are well-established to provide resilience to atherosclerotic cardiovascular disease [6, 7]. In addition to its role in reverse cholesterol transport, other vasoprotective functions of HDL include promoting endothelial nitric oxide (NO) synthase activity, reducing inflammation, and suppressing vascular adhesion molecule expression [8–12]. Importantly, aging and vascular disease can impair these functions [8, 13–15]. Using a novel model of 3D bioengineered human arteries, we recently identified additional HDL functions that are directly relevant to AD pathogenesis and involve the human cerebrovasculature. Specifically, we found that HDL circulated through the lumen of bioengineered human arteries prevents Aβ deposition within the bioengineered arterial wall, promotes Aβ transport from “brain” to “blood”, and attenuates Aβ-induced monocyte adhesion to endothelial cells both in monoculture and in engineered arteries [16, 17].

Considerable human data support a role for HDL in reducing AD risk. Two recent large genome wide association studies (GWAS) identified gene sets for lipoprotein metabolism and HDL components, namely *APOE*, *ABCA1*, *APOC1*, *APOM*, *APOA2*, *PON1*, *CLU*, *LCAT*, *CETP*, and *APOAI*, that were significantly associated with AD risk [18, 19]*.* Epidemiological studies, especially those that measure baseline high-density lipoprotein cholesterol (HDL-C) levels at middle age, have found significant associations between elevated plasma HDL-C levels and reduced AD risk and memory impairments [20–27]. Although other studies have found no relationship between HDL-C and cognitive impairment [28–33], many of these measured baseline HDL-C levels in aged subjects [32, 33] or had a relatively short follow-up [31, 33].

Studies in mice also support a protective role for HDL in AD pathogenesis. APP/PS1 mice lacking apoA-I (apoA-I^KO^) have more CAA [34] with no significant change in total amyloid burden [35, 36] or neuroinflammation [34]. Conversely, increasing HDL levels in Aβ overexpressing mice, either through transgenic overexpression or upon treatment with an apoA-I mimetic, resulted in reduced CAA [37], lower amyloid plaque load [38–41], and attenuated neuroinflammation [37–39, 41]. Others have administrated reconstituted HDL, recombinant apoA-I, and apoA-I mimetics to APP/PS1 or other AD model mice and similarly observed improvements in memory, neuroinflammation, and CAA, and some also observed reduced total soluble Aβ levels and Aβ deposition [39, 41–43]. In the present study, we used APP/PS1 mice deficient or hemizygous for apoA-I to extend these previous in vivo studies, confirm a clear role for apoA-I on cortical CAA and on cortical and hippocampal inflammation, and report a novel interaction among apoA-I, astrogliosis, and vascular and parenchymal amyloid.

## Materials and methods

### Animals

All procedures involving animals were approved by the Canadian Council of Animal Care and the University of British Columbia Committee on Animal Care. APP/PS1 mice (Jackson laboratories, B6.Cg-Tg(APPswe,PSEN1dE9)85Dbo/Mmjax, MMRRC stock no: 34832-JAX) on a C57Bl/6 background co-express two transgenes from the murine prion promoter: a chimeric mouse/human amyloid precursor protein (APP) cDNA containing the Swedish (K670 M/N671 L) mutations, and the human presenilin-1 (PS1) gene deleted for exon 9 [44]. APP/PS1 mice were first bred with apoA-I-deficient mice with targetted disruption in *Apoa1*(Jackson Laboratories, B6.129P2-*Apoa1*^*tm1Unc*^/J, Stock no: 002055, also on a C57Bl/6 background) to produce an F1 generation hemizygous for both *Apoa1*^*tm1Unc*^ and APP/PS1 transgenes. These animals where then backcrossed to apoA-I knockouts (homozygous for *Apoa1*^*tm1Unc*^) to produce F2 male and female mice of four genotypes: APP/PS1 mice hemizygous for *Apoa1* (APP/PS1 apoA-I^HEM^), APP/PS1 mice with complete *Apoa1* deficiency (APP/PS1 apoA-I^KO^), nontransgenic littermates hemizygous for *Apoa1* (WT apoA-I^HEM^), and nontransgenic littermates deficient in *Apoa1* (WT apoA-I^KO^). Although APP/PS1 mice on a pure C57Bl/6 background have an increased seizure risk, we selected this strain to maintain genetic homogeneity throughout this breeding strategy. Overall, 80% of the animals survived until the end of the experiment, with specific survival rates of 66% for APP/PS1 mice, 63% for APP/PS1 apoA-I^HEM^ mice, and 74% for APP/PS1 apoA-I^KO^ mice (Additional file 1: Figure S1a and b). While this breeding strategy was successful in generating *N* = 6–8 mice per group, insufficient animals survived to yield sex-specific cohorts. We therefore used mixed sexes, which is an acknowledged limitation of this study, yet tracking of individual animals throughout the study showed no clear sex bias throughout the results and justifies the use of pooled sexes.

### Tissue collection

Mice were fasted for 4 h prior to anesthetization by intraperitoneal injection of 20 mg/kg xylazine (Bayer) and 150 mg/kg ketamine (Bimeda-MTC). Blood was collected by cardiac puncture in ethylenediaminetetraacetic acid (EDTA)-containing syringes, centrifuged at 21,000*g* for 10 min at 4 °C, and the resulting plasma was stored at − 80 °C until use. Mice were then perfused for 6 min with ice-cold phosphate-buffered saline (PBS) containing 2500 U/L heparin at 6 mL/min. Brains were excised and bisected in the sagittal plane. A 2 × 5 mm piece of parietal cerebral cortex was removed and stored separately for RNA analysis. The piece of brain for RNA analysis and the remaining half-brain were snap-frozen on dry ice and stored at − 80 °C until use. The remaining half-brain was fixed in 4% paraformaldehyde (PFA) for 2 days at 4 °C followed by storage in PBS containing 30% sucrose and 0.1% sodium azide at 4 °C.

### Plasma lipid measurements

Plasma HDL cholesterol (HDL-C), low-density lipoprotein cholesterol (LDL-C), and total cholesterol levels were measured using Wako kits (Category no: 997-01301 for HDL-C, 993-00404 and 999-00504 for LDL-C, and 999-02601 for total cholesterol) as per the manufacturer’s directions adapted for 96-well microplates. Plasma samples were randomized, and the researcher performing the measurements was blinded to sample genotype. Plasma lipids were analyzed in *N* = 6–7 mice per genotype.

### Histology

Immunofluorescence triple co-staining was performed to visualize endothelial cells (cluster of differentiation 31; CD31), amyloid beta plaques (X-34), and activated astrocytes (glial fibrillary acidic protein; GFAP) as follows. Cryoprotected half brains were sectioned at 40 μm on a Leica CM3050 Research Cryostat and stored in PBS containing 0.1% sodium azide until staining. Three sections from each half-brain were selected from the anterior to posterior hippocampus at 200-μm intervals and mounted on Superfrost Plus slides. Antigen retrieval was performed with citrate buffer (10 mM citric acid, 0.05% Tween 20, pH 6.0) at 95 °C for 5 min followed by washing with PBS, tissue permeabilization with 0.25% Triton X-100 in PBS for 30 min, and blocking with 5% donkey serum and 1% BSA and 0.3% Triton X-100 in PBS for 60 min. Sections were incubated in primary antibodies for CD31 (Abcam, ab28364, 1:200 dilution), GFAP Alexa Fluor 488 (eBiosceince, 53-9892-80 1:400 dilution), or ICAM-1 (R&D, AF796, 1:50 dilution) in 5% donkey serum, 1% BSA, and 0.3% Triton X-100 in PBS overnight at 4 °C. After washing with PBS, sections were incubated with secondary antibodies for 60 min at room temperature. Secondary antibodies were as follows: goat anti-rabbit Alexa Fluor 594 antibody (Life Tech, A11012, 1:600 dilution) to detect CD31 on its own and when co-stained with GFAP, goat anti-rabbit Alexa Fluor 647 antibody (Life Tech, A27040, 1:400 dilution) to detect CD31 co-stained with ICAM-1, and donkey anti-goat Alexa Fluor 594 antibody (Life Tech, A-11058, 1:400 dilution) to detect ICAM-1. Sections were then washed with PBS. Amyloid staining was subsequently performed with X-34 (Sigma-Aldrich, SML 1954, 2 μM in PBS-T) for 20–30 min followed by washing with 40% ethanol in PBS then PBS alone. Coverslips were mounted onto slides in prolong antifade (Invitrogen, P36970), and slides were stored at 4 °C until imaging with an Axio Scan.Z1 (Zeiss). Brain sections were randomized, and the researcher performing the staining was blinded to sample genotype. The cellular localization of ICAM-1 expression was visualized using an LSM 880 confocal laser scanning microscope (Zeiss).

### Image analysis

Image analysis was performed with ImageJ (NIH) by a researcher blinded to sample genotype as illustrated in Additional file 1: Figure S2. Exported images for each channel were converted to 8-bit black and white images. The threshold for positive and negative pixels was manually determined for X-34 and GFAP images using histogram-based segmentation then applied to all of the images of that channel. Due to regional variation in CD31 staining, auto-local thresholding using the Bernsen method was performed on CD31 images. Cortical and hippocampal regions were manually selected and saved as “regions of interest” (ROIs) for each section. Total CD31, X-34, and GFAP-positive area was measured in the threshold segmented cortical and hippocampal ROIs and normalized to the total ROI area. Vascular GFAP was quantified as the GFAP-positive area associated with CD31 in each region whereby a mask of the segmented CD31 image for a section was created then applied to the segmented GFAP image of the same section. The GFAP-positive area within the CD31 mask was measured then normalized to the total CD31-positive area within the ROI. Plaque-associated GFAP was similarly quantified as the GFAP-positive area associated with X-34 in each region whereby a mask of the segmented X-34 image for a section was created then applied to the segmented GFAP image of the same section. The GFAP-positive area within the X-34 mask was measured then normalized to the total X-34-positive area within the ROI.

Total and vascular ICAM-1 quantification was performed using the same techniques as described above to measure total and vascular GFAP. Parenchymal ICAM-1-positive area was calculated as the difference between total and vascular ICAM-1-positive area in the cortical and hippocampal regions.

CAA was quantified using methods similar to that published in *Nature Protocols* by Wilcock et al. in 2006 [45], which has been used by many others [37, 46, 47] including in the study by Lefterov et al. using apoA-I-deficient APP/PS1 mice [34]. A mask containing areas of vascular amyloid was created by manually discriminating vascular amyloid from parenchymal plaques in segmented X-34 images based on morphology, as illustrated in Additional file 1: Figure S3. Identification of vascular versus parenchymal amyloid was performed on inverted images to ease visualization. These vascular amyloid masks were then applied back onto segmented X-34 images, and the percent positive area was measured and normalized to total area of the cortical ROI to give percent cortical CAA area. The X-34-positive area within the vascular amyloid mask was then used to create a new CAA mask. This CAA mask was then applied to a mask of vascular GFAP created as above. The vascular GFAP-positive area within the CAA mask was measured and normalized to CAA area to give the percent area of CAA associated with vascular GFAP.

The above analysis steps, excluding the drawing of cortical and hippocampal ROIs and vascular amyloid areas, were repeated for each section using an automated ImageJ macro. The full macro text for the imaging strategy is available in Additional file 2. Additional file 1: Figure S2 shows schematics illustrating the image analysis process. Analysis of vessel diameter and tortuosity was performed with Vesselucida 360 software (MBF Bioscience) using CD31 as the vessel marker. For amyloid staining, *N* = 4–5 animals per genotype were used, and for CD31, GFAP, and ICAM-1 staining, *N* = 5–6 animals per genotype were used.

### Protein extraction

Protein from half-brains was serially extracted to produce soluble and insoluble protein fractions. Half-brains were first homogenized with a Tissuemite homogenizer for 20 s in carbonate buffer (10 mmol/L carbonate, 50 mmol/L sodium chloride, cOmplete protease inhibitor tablet, pH 11.5) then sonicated. Homogenates were cleared by centrifugation at 12,500*g* for 45 min at 4 °C. The resulting supernatant was neutralized with 1.5 volumes Tris (1 mol/L Tris, pH 6.8) and labeled as the soluble fraction. The pellet was resuspended in 1 mL of guanidine hydrochloride (5 mol/L guanidine hydrochloride in 1 mol/L Tris, pH 8.0) by pipetting followed by rotation overnight at room temperature and labeled as the insoluble fraction. Soluble and insoluble extracts were stored at − 80 °C until use. Lysate protein concentrations were determined using the DC Protein assay kit (BioRad).

### Elisa

Human Aβ40 (KHB-3482, ThermoFisher, 1:20, 1:2500), human Aβ42 (KHB-3442, ThermoFisher, 1:40, 1:2500), and murine apoE (3752-1HP-2, ThermoFisher, 1:20, 1:200) levels were measured in carbonate soluble and guanidine hydrochloride insoluble half-brain lysates using commercial sandwich ELISA kits according to the manufacturer’s instructions. Murine GFAP (NS830, Millipore, 1:400), intercellular adhesion molecule 1 (ICAM-1) (ab100688, Abcam, 1:2), vascular cell adhesion molecule (VCAM-1) (ab100750, Abcam, 1:2000), platelet-derived growth factor receptor beta (PDGFRβ) (MBS919047, MyBioSource, 1:10), interleukin 1 beta (IL-1β) (K15245D, MesoScale Discovery, 1:2), and tau (K15121D, MesoScale Discovery, 1:50) levels were measured in carbonate soluble half-brain lysates using commercial sandwich or MesoScale Discovery ELISA kits according to the manufacturer’s instructions. Data points were interpolated from a standard curve using a 4-parameter nonlinear regression curve fitting and normalized to total soluble protein concentration. Brain lysates were randomized and the researcher performing the ELISAs was blinded to sample genotype. For apoE ELISA, *N* = 6 mice per genotype were used. For Aβ ELISA, *N* = 5–7 mice per genotype were used. For IL-1β, ICAM-1, VCAM-1, and PDGFRβ ELISA, *N* = 5–19 mice per genotype were used. For GFAP ELISA, *N* = 6–14 mice per genotype were used.

### RNA isolation and RT-qPCR

RNA was extracted from parietal cerebral cortices using Trizol (Invitrogen) and treated with DNaseI (Life Technologies) prior to cDNA synthesis. cDNA was generated using the Taqman Reverse Transcription Kit (ThermoFisher, N8080234). RT-qPCR was performed using the Light Cycler 96 Real-Time PCR System (Roche) and FastStart Essential DNA Green Master Mix (Roche). Expression of murine *Abca1* (Fwd: CTGACCTATGTGCTGCCGTA; Rev.: GAGCCGGTCATCAATCTCAT) and murine *Il1b* (Fwd: TTGACGGACCCCAAAAGA; Rev.: CAGCTTCTCCACAGAGCCACA) were normalized to murine β-actin (Fwd: ACGGCCAGGTCATCACTATTG; Rev.: CAAGAAGGAAGGCTGGAAAAG). RNA homogenates were randomized, and the researcher performing the qRT-PCR was blinded to sample genotype. For *Apoe* and *Il1b* mRNA analyses, *N* = 7–12 mice per genotype were used.

### Contextual and cued fear conditioning

#### Training

During training, mice were placed in an illuminated compartment of a shuttle box (Med Associates Inc., St. Albans, VT) and allowed to habituate to the internal testing environment for 120 s. After the habituation period, an 80-dB auditory cue was played for 30 s. During the last 2 s of the auditory cue, a mild foot shock (0.3 mA) was administered. Shocked mice were left undisturbed in the testing chamber for an inter-trial interval (ITI) of 60 s, after which mice were presented with a second identical tone, which was called the shock trial. After the second tone, shocked mice were again left undisturbed in the testing chamber for 60 s.

#### Context testing

Twenty-four hours after training, mice were tested for their ability to remember the context in which they received the foot shock. Mice were placed in the same illuminated compartment and observed for the presence/absence of a freezing response over 5 min. During this time, mice were not exposed to the tone or shock and the percent freezing time was measured using the ANY-Maze system (Stoelting Co., Wood Dale, IL). Contextual testing included *N* = 7–23 mice per genotype.

#### Cued testing

One hour following context testing, mice were tested for their ability to remember the 80-dB auditory cue presented during the training session. Testing was conducted in an environment different from the training and context testing session (darkened compartment, false floor placed over the steel rods of the shuttle box, different cleaning solution). Mice were placed in the darkened compartment and allowed to habituate for 120 s, followed by the 80 dB auditory cue for 120 s. After the tone, mice were left undisturbed in the testing chamber for 60 s and percent freezing time was measured using the ANY-Maze system. Cued testing included *N* = 7–23 mice per genotype.

### Statistical analysis

All statistical analyses were performed with GraphPad Prism 7; *p* values < 0.05 were considered statistically significant. For the analysis of all non-amyloid data, statistical comparisons were made by two-way ANOVA considering APP/PS1 genotype as one factor and apoA-I genotype as a second factor (omnibus analysis) followed by Sidak’s multiple comparisons test if significant factor or interaction effects were observed. Analysis of Aβ levels, amyloid plaque area, and CAA were performed with unpaired *t* test or Mann-Whitney test for parametric and non-parametric data, respectively.

## Results

### Loss of apoA-I significantly reduces plasma total and HDL cholesterol levels

**Fig. 1:**
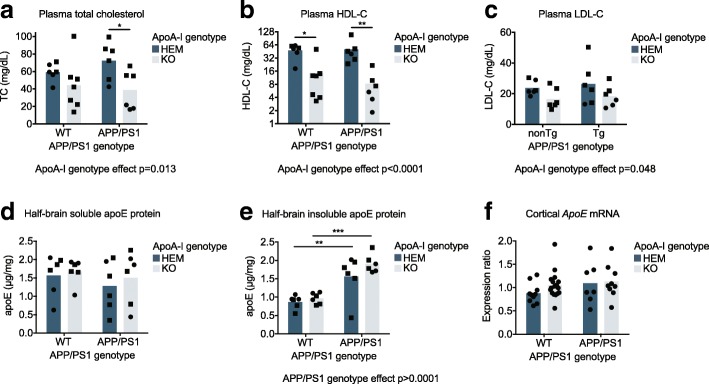
Loss of apoA-I significantly reduced plasma cholesterol levels. Plasma levels of (**a**) total cholesterol, (**b**) HDL-C, and (**c**) LDL-C were measured with commercially available kits. ApoE protein in (**d**) soluble and (**e**) insoluble half-brain homogenates was measured by ELISA. **f** *Apoe* mRNA expression was measured in the parietal cerebral cortex by qRT-PCR. Points represent individual mice and bars represent mean values. Circles represent female mice, and squares represent male mice. Omnibus analyses of apoA-I and APP/PS1 genotype effects by two-way ANOVA are displayed as exact *p* values below graphs. Sidak’s multiple comparisons test results are displayed within graphs as *p < 0.05, **p < 0.01, and ***p < 0.0001. For plasma lipid and brain protein analysis, *N* = 6–7 mice per genotype; for mRNA analysis *N* = 7–17 mice per genotype were used. apoA-I, apolipoprotein A-I; TC, total cholesterol; HDL-C, high-density lipoprotein cholesterol; LDL-C, low-density lipoprotein cholesterol; HEM, hemizygous apoA-I genotype; KO, knockout apoA-I genotype; WT, wildtype APP/PS1 genotype; APP/PS1, transgenic APP/PS1 genotype; apoE, apolipoprotein E

By omnibus two-way ANOVA, apoA-I deficiency significantly reduced plasma total cholesterol (*p* = 0.013), HDL-C (*p* < 0.0001), and LDL-C levels (*p* = 0.048) (Fig. 1a–c) relative to apoA-I^HEM^ mice, as previously reported [48, 49]. HDL-C was the cholesterol pool most affected by the complete loss of apoA-I with reductions from 48.3 mg/dL to 14.6 mg/dL (*p* = 0.011) in WT apoA-I^KO^ vs. WT apoA-I^HEM^ mice and from 50.1 mg/dL to 8.3 mg/dL (*p* = 0.003) in APP/PS1 apoA-I^KO^ vs. APP/PS1 apoA-I^HEM^ mice. Plasma cholesterol pools were not affected by APP/PS1 genotype. ApoA-I genotype did not affect soluble (Fig. 1d) or insoluble (Fig. 1e) brain apoE protein levels or cortical *Apoe* mRNA expression (Fig. 1f), although insoluble apoE protein levels in the brain were significantly higher in APP/PS1 mice compared to WT controls overall (*p* < 0.0001) (Fig. 1e).

### ApoA-I deficiency increases cortical parenchymal and vascular amyloid burden of APP/PS1 mice

**Fig. 2:**
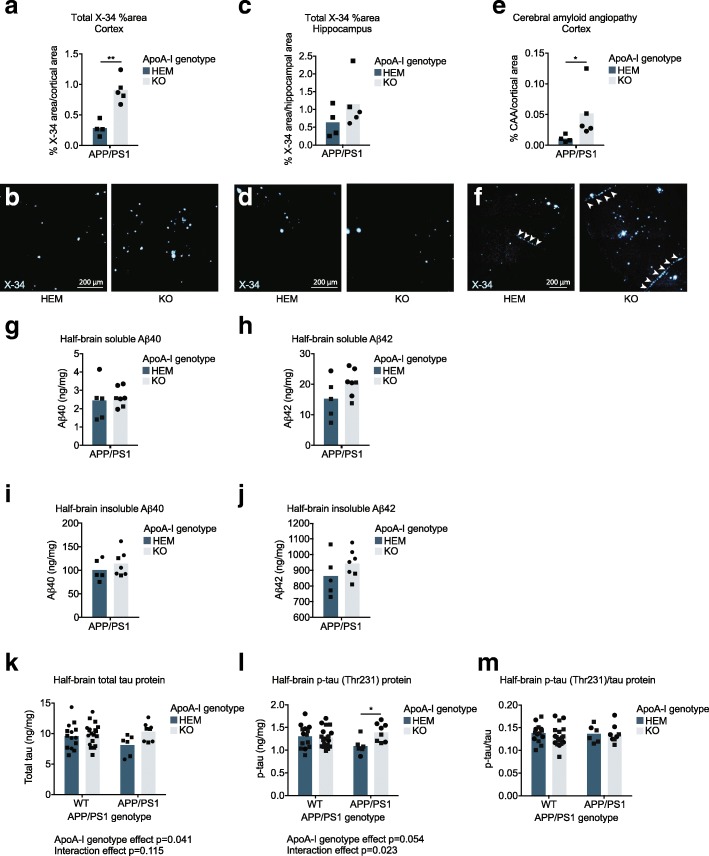
Cortical amyloid plaques and vascular Aβ deposition were increased the absence of apoA-I. Total (**a**, **b**) cortical and (**c**, **d**) hippocampal area occupied by amyloid deposits and (**e**, **f**) CAA area were evaluated by staining fixed cryosections with X-34. Representative images are below graphs. **g** Soluble Aβ40, **h** soluble Aβ42, **i** insoluble Aβ40, and **j** insoluble Aβ42 protein levels were measured in half-brain homogenates by ELISA. **k** Total tau and **l** phosphorylated tau proteins were measured by ELISA and used to calculate the (**m**) phosphorylated tau/total tau ratio. All analytes were normalized to homogenate total protein concentration. Points represent individual mice, and bars represent mean values. Circles represent female mice, and squares represent male mice. Arrowheads in **f** indicate areas of CAA. Results of unpaired *t* test (**a**) and Mann-Whitney test (**e**) are displayed within graphs as *p < 0.05 and **p < 0.01. Omnibus analyses of apoA-I and APP/PS1 genotype effects by two-way ANOVA are displayed as exact *p* values below graphs (**k**, **l**). Sidak’s multiple comparisons test results (**l**) are displayed within the graph as *p < 0.05. For amyloid staining, *N* = 4–5 mice per genotype were used; for Aβ ELISA, *N* = 5–7 mice per genotype were used; for tau and p-tau ELISA, *N* = 6–19 mice per genotype were used. apoA-I, apolipoprotein A-I; HEM, hemizygous apoA-I genotype; KO, knockout apoA-I genotype; WT, wildtype APP/PS1 genotype; APP/PS1, transgenic APP/PS1 genotype; CAA, cerebral amyloid angiopathy; Aβ, amyloid beta; p-tau, phosphorylated tau; X-34, amyloid stain

Histological examination of X-34-stained sections showed that apoA-I deficiency led to significantly greater amyloid burden in the cortex, an increase from a mean of 0.28% to 0.91% cortical area (Fig. 2a, b) (*p* = 0.001 by unpaired *t* test), but not in the hippocampus (Fig. 2c, d) of APP/PS1 mice. Consistent with a previous study [34], we also observed significantly increased cortical CAA from a mean of 0.01% to 0.05% cortical area in APP/PS1 apoA-I^KO^ compared to APP/PS1 apoA-I^HEM^ mice (*p* = 0.016 by Mann-Whitney test) (Fig. 2e, f). CAA was not observed in the hippocampus in any mice. These observations suggest that apoA-I protects against Aβ deposition in the walls of cortical arteries, which may in turn reduce parenchymal amyloid deposition in the surrounding cortex.

Biochemical analysis of total soluble and insoluble Aβ40 and Aβ42 pools in half-brain homogenates revealed no differences by apoA-I genotype, although soluble Aβ40, insoluble Aβ40, and insoluble Aβ42 were all very slightly and non-significantly increased in APP/PS1 apoA-I^KO^ vs. APP/PS1 apoA-I^HEM^ mice (Fig. 2g–j). The lack of a significant effect of apoA-I genotype on Aβ levels, despite the significant increase in cortical amyloid deposition, may be due to a region-specific effect of apoA-I on amyloid that is not detectable in crude half-brain homogenates. Indeed, immunofluorescence analysis (described above) confirms that apoA-I genotype influences amyloid plaque burden in the cortex but not in the hippocampus.

Although no significant differences in tau or p-tau were observed by APP/PS1 genotype, total tau levels (Fig. 2k) were significantly higher and p-tau levels (Fig. 2l) tended to be higher in apoA-I^KO^ mice compared to apoA-I^HEM^ mice (*p* = 0.041 and *p* = 0.054, respectively, by omnibus two-way ANOVA). ApoA-I genotype significantly interacted with APP/PS1 genotype with respect to p-tau (*p* = 0.023). Post-hoc Sidak’s multiple comparison tests revealed a nearly significant increase in total tau in APP/PS1 apoA-I^KO^ vs. APP/PS1 apoA-I^HEM^ mice (*p* = 0.063) and a significant increase in p-tau from 1.09 ng/mg in APP/PS1 apoA-I^KO^ to 1.40 ng/mg in APP/PS1 apoA-I^HEM^ (*p* = 0.026). Levels of p-tau showed a trend towards a reduction in APP/PS1 apoA-I^HEM^ vs. WT apoA-I^HEM^ mice (*p* = 0.099 by Sidak’s multiple comparisons test). The total tau to p-tau ratio was unaffected by loss of apoA-I (Fig. 2m).

### ApoA-I deficiency increases cortical levels of pro-inflammatory protein and mRNA markers in APP/PS1 mice

**Fig. 3:**
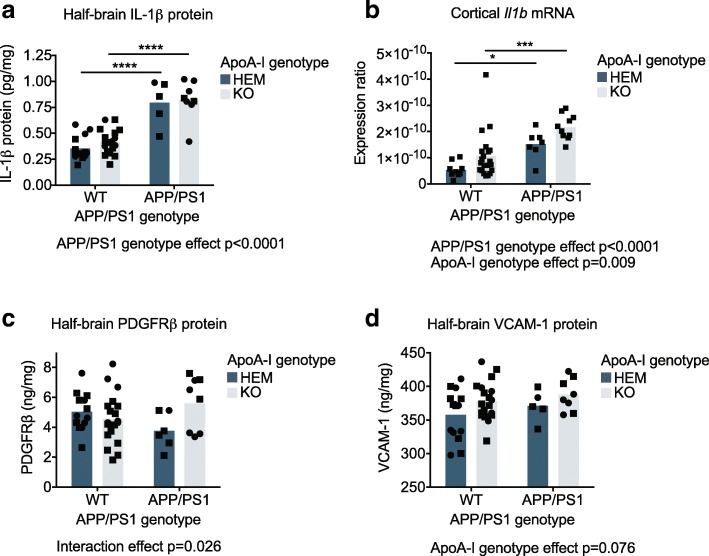
Cortical levels of pro-inflammatory protein and mRNA markers were increased in the absence of apoA-I. **a** IL-1β, **c** PDGFRβ, and **d** VCAM-1 protein levels were measured by ELISA in soluble half-brain homogenates; values were normalized to total protein concentration in the homogenates. **b** *Il1b* mRNA expression was measured in the cortex by qRT-PCR and normalized to *β-actin* expression. Points represent individual mice, and bars represent mean values. Circles represent female mice, and squares represent male mice. Omnibus analyses of apoA-I and APP/PS1 genotype effects by two-way ANOVA are displayed as exact *p* values below graphs. Sidak’s multiple comparisons test results are displayed within graphs as *p < 0.05, ***p < 0.001, and ****p < 0.0001. For ELISA, *N* = 5–19 mice per genotype were used; for mRNA, *N* = 7–21 mice per genotype were used. apoA-I, apolipoprotein A-I; HEM, hemizygous apoA-I genotype; KO, knockout apoA-I genotype; WT, wildtype APP/PS1 genotype; APP/PS1, transgenic APP/PS1 genotype; IL-1β, interleukin 1 beta; VCAM-1, vascular cell adhesion molecule 1; PDGFRβ, platelet-derived growth factor receptor beta

IL-1β protein levels in half-brain homogenates were elevated in APP/PS1 compared to WT mice from a mean of 0.36 pg/mg and 0.42 pg/mg in WT apoA-I^HEM^ and apoA-I^KO^, respectively, to 0.79 pg/mg and 0.82 pg/mg in APP/PS1 apoA-I^HEM^ and apoA-I^KO^, respectively, (*p* < 0.0001 by omnibus two-way ANOVA). IL-1β protein levels were not affected by apoA-I genotype (Fig. 3a). However, cortical *Il1b* mRNA levels were significantly elevated overall in APP/PS1 compared to WT mice (*p* < 0.0001 omnibus by two-way ANOVA) and in apoA-I^KO^ vs. apoA-I^HEM^ mice (*p* = 0.009 by omnibus two-way ANOVA) (Fig. 3b), where the mean expression ratio was 5.37e-11 in WT apoA-I^HEM^, 1.06e-10 in WT apoA-I^KO^, 1.51e-10 in APP/PS1 apoA-I^HEM^, and 2.17e-10 in APP/PS1 apoA-I^KO^. The discrepancy between IL-1β protein and mRNA observations may be due to both the lower limit of sensitivity for the protein assay and regional selectivity. Brain protein and mRNA expression of TNFα and IL-6 were also measured but were all below limits of detection (data not shown).

We next investigated markers of inflammation expressed specifically by cells of the blood-brain barrier. PDGFRβ is a pericyte marker that is elevated in some cases of neuroinflammation [50]. We observed a significant interaction effect of APP/PS1 and apoA-I genotypes (*p* = 0.026 by omnibus two-way ANOVA) on PDGFRβ protein levels in the brain, where PDGFRβ protein tended to be elevated only in APP/PS1 apoA-I^KO^ mice compared to APP/PS1 apoA-I^HEM^ mice (*p* = 0.065 by Sidak’s multiple comparisons test) (Fig. 3c). Mean PDGFRβ levels were 5.03 ng/mg in WT apoA-I^HEM^, 4.56 ng/mg in WT apoA-I^KO^, 3.76 ng/mg in APP/PS1 apoA-I^HEM^, and 5.61 ng/mg in APP/PS1 apoA-I^KO^. To evaluate endothelial cell-specific inflammation, we measured protein levels of VCAM-1 in the brain. VCAM-1 is expressed on the cell surface of endothelial cells and assists in the adhesion of circulating immune cells to the vessel wall [11]. We found that VCAM-1 tended to be elevated in apoA-I^KO^ mice compared to apoA-I^HEM^ mice overall (*p* = 0.076 by omnibus two-way ANOVA) independent of APP/PS1 genotype (Fig. 3d). Together, these data suggest that loss of apoA-I can exacerbate neuroinflammation in APP/PS1 mice, particularly in cell types associated with the cerebrovasculature.

### ApoA-I deficiency increases total ICAM-1 protein levels in the brain, total ICAM-1 positive area in the hippocampus, and vascular ICAM-1 levels in the cortex and hippocampus of APP/PS1 mice

**Fig. 4:**
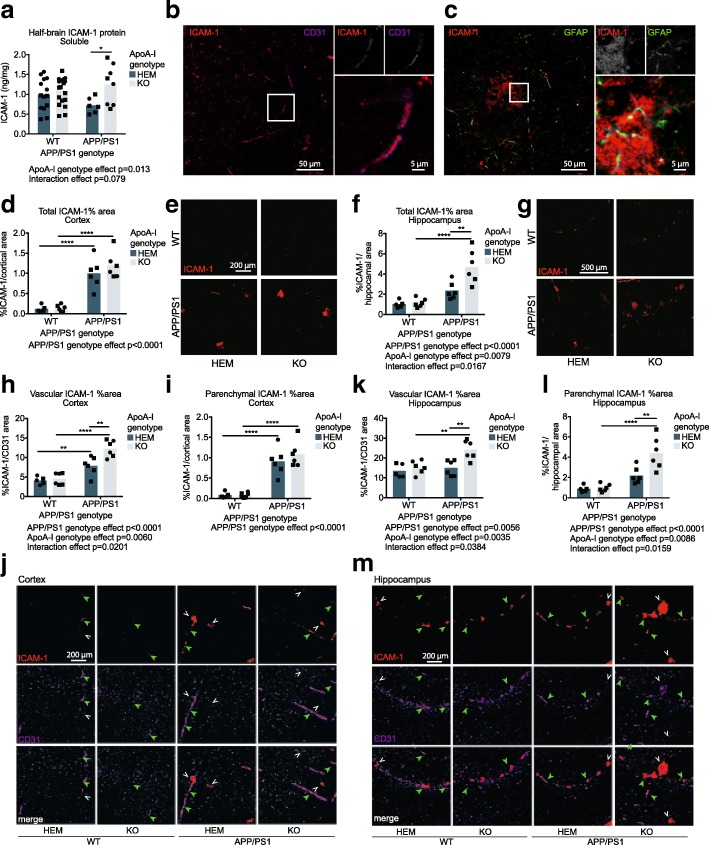
Absence of apoA-I increases ICAM-1 protein levels, hippocampal parenchymal ICAM-1 levels, and both cortical and hippocampal endothelial ICAM-1 levels. **a** Total ICAM-1 protein in soluble half-brain homogenates was measured by ELISA. Confocal microscopy was used to (**b**) identify co-localization of ICAM-1 with the endothelial cell marker CD31 and (**c**) evaluate the co-localization of some ICAM-1 in the brain parenchyma with GFAP-positive astrocytes. Total ICAM-1 staining area was visualized by immunofluorescence in cortical (**d**, **e**) and hippocampal (**f**, **g**) regions and positive staining area was normalized to total region area (**h**, **j**, **k**, **m**). Vascular-specific and parenchymal ICAM-1 expression was visualized using immunofluorescence and shows association of GFAP with CD31 in (**h**–**j**) cortical and (**k**–**m**) hippocampal regions, where positive co-stained area was normalized to total CD31-positive area. Representative images for immunofluorescent data are below the graphs. Points represent individual mice, and bars represent mean values. Circles represent female mice, and squares represent male mice. Omnibus analyses of apoA-I and APP/PS1 genotype effects by two-way ANOVA are displayed as exact *p* values below graphs. Sidak’s multiple comparisons test results are displayed within graphs as *p < 0.05, **p < 0.01, ***p < 0.001, and ****p < 0.0001. For ELISA, *N* = 6–19 mice per genotype were used. apoA-I, apolipoprotein A-I; HEM, hemizygous apoA-I genotype; KO, knockout apoA-I genotype; WT, wildtype APP/PS1 genotype; APP/PS1, transgenic APP/PS1 genotype; ICAM-1, intercellular adhesion molecule 1; GFAP, glial fibrillary acidic protein; CD31, cluster of differentiation 31. Green closed arrowheads indicate examples of vascular ICAM-1, and white open arrowheads indicate examples of parenchymal ICAM-1

ICAM-1 is another endothelial cell adhesion molecule that functions similarly to VCAM-1. ICAM-1 can also be expressed in other cells such as astrocytes and microglia [51–54]. We observed an overall effect of apoA-I genotype on brain ICAM-1 levels with increased levels in apoA-I^KO^ mice (*p* = 0.013 by omnibus two-way ANOVA), a specific increase in APP/PS1 apoA-I^KO^ mice compared to APP/PS1 apoA-I^HEM^ mice from a mean of 0.72 ng/mg to 1.25 ng/mg (*p* = 0.023 by Sidak’s multiple comparisons test), and a trend towards a significant interaction effect between apoA-I and APP/PS1 genotypes (*p* = 0.079 by omnibus two-way ANOVA) (Fig. 4a). Confocal imaging confirmed co-localization of ICAM-1 with CD31 (Fig. 4b) and showed that only some of the parenchymal ICAM-1 was co-localized with GFAP-positive astrocytes, suggesting expression by other cell types (Fig. 4c).

The effect of apoA-I genotype on total brain ICAM-1 levels was found to be region specific using immunofluorescence. Although we observed no significant differences in total cortical ICAM-1 staining by apoA-I genotype (Fig. 4b), total ICAM-1-positive area was increased in APP/PS1 apoA-I^KO^ compared to APP/PS1 apoA-I^HEM^ in the hippocampus, from 2.34% of hippocampal area to 4.67% (*p* = 0.001 by Sidak’s multiple comparisons test) (Fig. 4c). Interestingly, vascular-specific ICAM-1, measured as the ICAM-1-positive area associated with CD31, was significantly elevated in both cortex (Fig. 4d, e) and hippocampus (Fig. 4f, g) in APP/PS1 apoA-I^KO^ compared to APP/PS1 apoA-I^HEM^. Cortical vascular ICAM-1 levels were 7.87% and 12.26% of CD31-positive area in APP/PS1 apoA-I^HEM^ and APP/PS1 apoA-I^KO^, respectively (*p* = 0.001 by Sidak’s multiple comparisons test) (Fig. 4h, j) and hippocampal levels were 15.02% and 24.19% (*p* = 0.001 by Sidak’s multiple comparisons test) (Fig. 4k,m). Although no differences in parenchymal ICAM-1-positive area were observed in the absence of apoA-I (Fig. 4i, j), parenchymal ICAM-1-positive area was increased in the hippocampus from 2.19% of hippocampal area in APP/PS1 apoA-I^HEM^ to 4.41% in APP/PS1 apoA-I^KO^ (*p* = 0.001 by Sidak’s multiple comparisons test) (Fig. 4l, m).

### ApoA-I deficiency exacerbates Aβ-mediated increases in total and cerebrovascular GFAP levels in both cortex and hippocampus

**Fig. 5:**
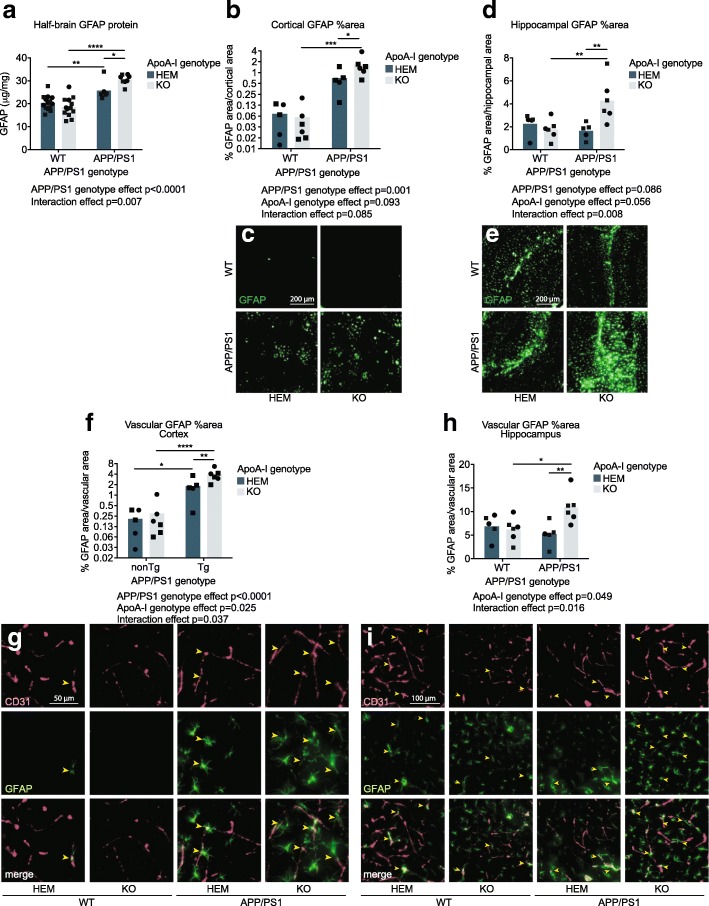
Loss of apoA-I increased the reactivity of astrocytes associated with the cerebrovasculature. **a** Total GFAP protein levels were measured in soluble half-brain homogenates by ELISA, and values were normalized to total protein concentration in the homogenates. GFAP staining area was visualized by immunofluorescence in cortical (**b**, **c**) and hippocampal (**d**, **e**) regions, and positive staining area was normalized to total region area. Vascular-specific GFAP expression was visualized using immunofluorescence and observing the association of GFAP with CD31 in cortical (**f**, **g**) and hippocampal (**h**, **i**) regions, positive co-stained area was normalized to total CD31-positive area. Representative images for immunofluorescent data are below graphs. Points represent individual mice, and bars represent mean values. Circles represent female mice, and squares represent male mice. Yellow arrowheads indicate examples of areas of CD31-associated GFAP in the cortex (**g**) and hippocampus (**i**) that are quantified in (**f**) and (**h**), respectively. Omnibus analyses of apoA-I and APP/PS1 genotype effects by two-way ANOVA are displayed as exact *p* values below graphs. Sidak’s multiple comparisons test results are displayed within graphs as *p < 0.05, **p < 0.01, ***p < 0.001, and ****p < 0.0001. For GFAP ELISA, *N* = 6–14 mice per genotype were used; for GFAP total and vascular staining, *N* = 5–6 mice per genotype were used. apoA-I, apolipoprotein A-I; HEM, hemizygous apoA-I genotype; KO, knockout apoA-I genotype; WT, wildtype APP/PS1 genotype; APP/PS1, transgenic APP/PS1 genotype; GFAP, glial fibrillary acidic protein; CD31, cluster of differentiation 31

We observed a significant interaction effect between APP/PS1 and apoA-I genotypes on total GFAP protein levels in half-brain homogenates (*p* = 0.007 by omnibus two-way ANOVA). GFAP was elevated both in APP/PS1 mice compared to WT controls overall (*p* < 0.0001 by omnibus two-way ANOVA) and in APP/PS1 apoA-I^KO^ compared to APP/PS1 apoA-I^HEM^ mice (*p* = 0.030 by post-hoc Sidak’s multiple comparisons test) (Fig. 5a). Mean GFAP protein levels were 20.41 μg/mg in WT apoA-I^HEM^, 18.55 μg/mg in WT apoA-I^KO^, 25.75 μg/mg in APP/PS1 apoA-I^HEM^, and 30.59 μg/mg in APP/PS1 apoA-I^KO^. Immunofluorescent staining for GFAP confirmed the biochemical total protein data, with an additive effect of Aβ overexpression and apoA-I loss on GFAP staining area in the cortex (*p* = 0.001 for APP/PS1 vs. WT omnibus two-way ANOVA analysis; *p* = 0.001 for APP/PS1 apoA-I^KO^ vs. WT apoA-I^KO^; and *p* = 0.040 for APP/PS1 apoA-I^KO^ vs. APP/PS1 apoA-I^HEM^ by post-hoc Sidak’s multiple comparisons test) (Fig. 5b, c) and a synergistic effect in the hippocampus (*p* = 0.008 interaction effect by omnibus two-way ANOVA; *p* = 0.004 for APP/PS1 apoA-I^KO^ vs. WT apoA-I^KO^; and *p* = 0.004 for APP/PS1 apoA-I^KO^ vs. APP/PS1 apoA-I^HEM^ by Sidak’s multiple comparisons test) (Fig. 5d, e). Cortical GFAP-positive area increased from 0.07% and 0.06% in WT apoA-I^HEM^ and apoA-I^KO^, respectively, to 0.70% in APP/PS1 apoA-I^HEM^ and 1.66% in APP/PS1 apoA-I^KO^. In the hippocampus, GFAP-positive area was 2.27%, 1.77%, 1.65% and 4.28% in WT apoA-I^HEM^, WT apoA-I^KO^, APP/PS1 apoA-I^HEM^, and APP/PS1 apoA-I^KO^ respectively.

We next used immunofluorescence to examine the association of CD31 and GFAP and observed a robust and statistically significant increase in the cerebrovascular area associated with GFAP in both the cortex and hippocampus of APP/PS1 apoA-I^KO^ mice compared to APP/PS1 apoA-I^HEM^ mice (*p* = 0.008, *p* = 0.007, respectively, by Sidak’s multiple comparisons test) (Fig. 5f–i). In addition, significant elevations were observed overall in APP/PS1 compared to WT mice (*p* < 0.0001 in the cortex by omnibus two-way ANOVA) and in apoA-I^KO^ compared to apoA-I^HEM^ mice (*p* = 0.025 and *p* = 0.049 in the cortex and hippocampus, respectively, by omnibus two-way ANOVA). Mean cortical vascular GFAP levels were elevated from 0.21% and 0.30% of CD31-positive vascular area in WT apoA-I^HEM^ and apoA-I^KO^, respectively, to 1.80% in APP/PS1 apoA-I^HEM^ mice and 3.86% in APP/PS1 apoA-I^KO^. In the hippocampus, mean vascular GFAP levels increased from 6.86%, 6.20%, and 5.20% in WT apoA-I^HEM^, WT apoA-I^KO^, and APP/PS1 apoA-I^HEM^, respectively, to 10.86% in APP/PS1 apoA-I^KO^. Strikingly, the elevation in vascular GFAP in apoA-I^KO^ mice was statistically more robust than the changes in total GFAP-positive area in both the cortex and the hippocampus. The total vascular area vessel thickness, and vessel tortuosity as defined by CD31 staining were all unchanged by apoA-I genotype in the cortex and the hippocampus (Additional file 1: Figure S4a-h).

**Fig. 6:**
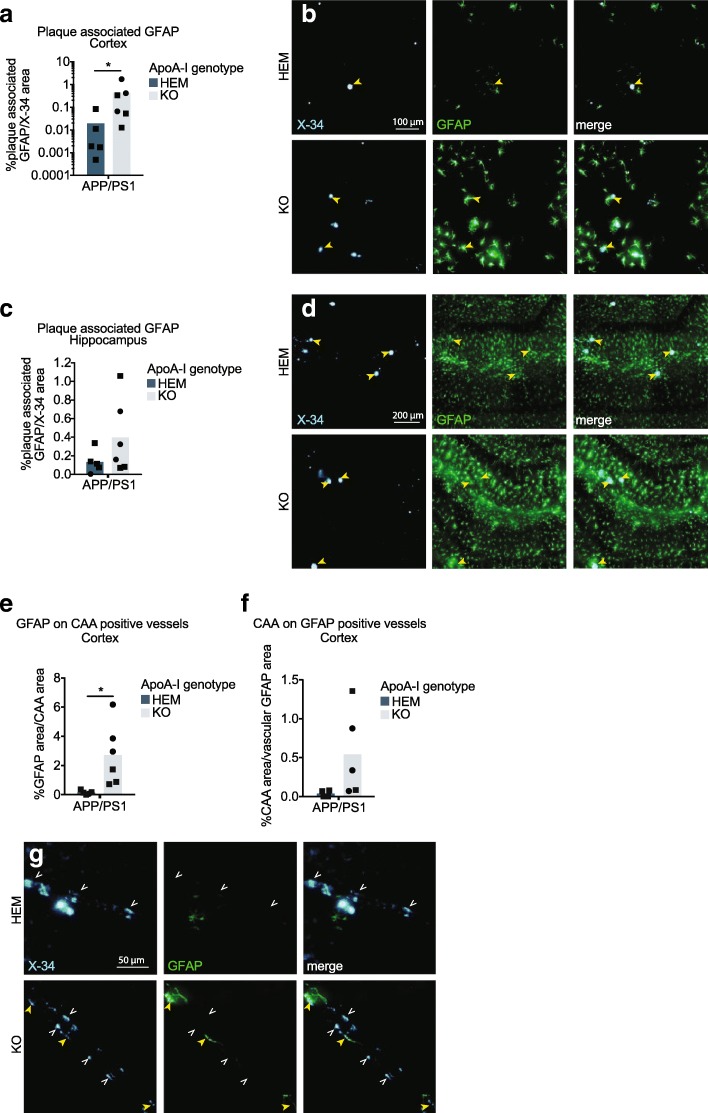
Loss of apoA-I increases astrocyte reactivity to Aβ plaques and vascular Aβ deposits. GFAP associated with Aβ plaques in the cortex (**a**, **b**) and hippocampus (**c**, **d**) was visualized with immunofluorescence and normalized to total area of the region. The association of vascular-specific GFAP (GFAP co-localized with CD31) with CAA was visualized using immunofluorescence and normalized to total cortical CAA area (**e**, **g**) and total vascular GFAP area (**f**, **g**) in the cortex. Representative images are beside (**a**, **c**) or below (**e**, **f**) graphs. Points represent individual mice, and bars represent mean values. Circles represent female mice, and squares represent male mice. Yellow closed arrowheads () indicate examples of areas of plaque-associated or CAA-associated GFAP, and white open arrowheads () indicate examples areas of plaque or CAA not associated with GFAP. Arrowheads in (**g**) indicate areas of CAA and vascular GFAP. Results of Mann-Whitney test (**a**) and unpaired *t* test (**e**) are displayed within graphs as *p < 0.05. For plaque and CAA area associated with GFAP, *N* = 5–6 mice per genotype were used; for CAA area associated with vascular GFAP, *N* = 4–5 mice per genotype were used. apoA-I, apolipoprotein A-I; HEM, hemizygous apoA-I genotype; KO, knockout apoA-I genotype; APP/PS1, transgenic APP/PS1 genotype; GFAP, glial fibrillary acidic protein; CD31, cluster of differentiation 31; X-34, amyloid stain; CAA, cerebral amyloid angiopathy

A closer examination of APP/PS1 mice revealed significantly greater levels of cortical plaque-associated GFAP in APP/PS1 apoA-I^KO^ compared to APP/PS1 apoA-I^HEM^, which increased from a mean of 0.02% to 0.51% of plaque area (*p* = 0.030 by Mann-Whitney test) (Fig. 5a, b), suggesting that apoA-I deficiency promotes astrocyte reactivity to Aβ plaques. Parenchymal plaque-associated GFAP was not significantly affected by apoA-I genotype in hippocampus (Fig. 6c, d). We also investigated whether astrocyte reactivity was affected by CAA, and observed a significant elevation of GFAP area on CAA-positive vessels from 0.17% of CAA area on average in APP/PS1 apoA-I^KO^ mice compared to 2.67% in APP/PS1 apoA-I^HEM^ controls (*p* = 0.023 by unpaired *t* test) (Fig. 6e, g). Notably, this effect was larger in magnitude than the increases in either CAA levels or vascular GFAP expression alone in APP/PS1 apoA-I^KO^ mice compared to APP/PS1 apoA-I^HEM^ mice. Finally, we observed a trend towards increased CAA in the areas of vascular GFAP expression 0.04% of vascular GFAP area on average in APP/PS1 apoA-I^KO^ mice compared to 0.54% in APP/PS1 apoA-I^HEM^ controls (*p* = 0.116 by unpaired *t* test) (Fig. 6f, g). Taken together, our data support an interaction among Aβ, astrogliosis, and apoA-I.

### ApoA-I deficiency does not significantly affect contextual or cued fear memory

Others have observed that genetically altering apoA-I levels in APP/PS1 mice affects spatial learning and memory, with increased apoA-I improving performance and loss of apoA-I worsening performance in the Morris Water Maze [34, 37]. We performed contextual and cued fear conditioning tests to evaluate fear memory (Additional file 1: Figure S5a and b). No significant genotype effects were observed with either test despite a trend towards reduced performance in both APP/PS1 vs. WT mice (*p* = 0.109) and in apoA-I^KO^ vs. apoA-I^HEM^ mice (*p* = 0.077) overall in the contextual fear conditioning test. Notably, the cohort of mice available for behavior testing was relatively small, particularly for the APP/PS1 apoA-I^KO^ group (*n* = 7); therefore, our behavior analysis was likely underpowered.

## Discussion

A better understanding of the vascular contributions to AD may reveal attractive therapeutic targets that act systemically and therefore may not necessarily need to cross the blood-brain barrier to have beneficial effects on the brain. Circulating factors such as HDL, which has multiple vasoprotective properties, could promote cerebrovascular health by acting on the luminal side of cerebral vessels. We therefore aimed to confirm and extend previous findings by us and others on the role of apoA-I on AD pathology in mice. In agreement with Lefterov et. al. [34], here, we observed that complete loss of apoA-I increases CAA in APP/PS1 mice. We also show for the first time that eliminating apoA-I in APP/PS1 mice increases total cortical Aβ deposition and astrogliosis in both the cortex and hippocampus. Additionally, we observed that loss of apoA-I increased several markers neuroinflammation and vascular inflammation within the brain. Specifically, brain levels of *Il1b* mRNA, ICAM-1 protein, PDGFRβ protein, and GFAP protein were elevated in the absence of apoA-I. Furthermore, apoA-I^KO^ mice showed elevated levels of ICAM-1 on endothelial cells and GFAP-positive astrocytes in direct contact with the cerebrovasculature in the cortex and hippocampus. While Lefterov et al. did not observe changes in amyloid pathology or neuroinflammation in apoA-I^KO^ mice, other groups have increased apoA-I levels in APP/PS1 mice using transgenic overexpression of apoA-I or by injecting apoA-I mimetics and observed reduced cerebral amyloid and astrogliosis [37, 39–41, 43]. Finally, we found that astrocyte reactivity to both parenchymal and vascular amyloid was increased in apoA-I-deficient mice. Importantly, the previous study by Lefterov et al. using compared APP/PS1 apoA-I^KO^ mice to APP/PS1 apoA-I^WT^ mice whereas the current work compares APP/PS1 apoA-I^KO^ mice to APP/PS1 apoA-I^HEM^ mice. Although others have shown that there is a greater difference in plasma apoA-I levels between apoA-I^KO^ and apoA-I^HEM^ mice compared to apoA-I^HEM^ and apoA-I^WT^ mice, the differences in genotypes used in each of these studies may contribute to some of the differences in the findings. Nonetheless, our observations support the hypothesis that plasma HDL acts to protect cerebral vessels not only from Aβ deposition but also from Aβ-induced astrocyte reactivity.

The mechanisms by which circulating HDL can reduce amyloid pathology and neuroinflammation are not completely understood but may include effects of apoA-I within the brain and effects of HDL/apoA-I from the periphery. ApoA-I has been shown to enter the brain parenchyma of mice via the blood-CSF barrier at the choroid plexus [55]. ApoA-I that has crossed into the brain may thus have direct anti-inflammatory effects on astrocytes consistent with apoA-I’s anti-inflammatory effects on other cell types [56, 57] or may reduce Aβ-induced inflammation by inhibiting Aβ fibrillization as has been observed in vitro [17]. Alternatively, HDL may exert its effects on the brain indirectly through actions on the cerebrovasculature, where it would act from the lumen of cerebral vessels on cells of the neurovascular unit without crossing into the brain parenchyma. The latter explanation appears more plausible for several reasons. First, the levels of apoA-I observed within the brain are low compared to the levels found circulating in the blood (ELISA and immunoblot levels of apoA-I in brain interstitial fluid are < 0.005% of plasma levels) [55]. Second, HDL has multiple vasoprotective properties including anti-inflammatory activity, modulation of vessel tone, and promotion of endothelial cell repair [58]. Third, HDL has Aβ-specific vasoprotective functions in 3D bioengineered human arteries including preventing vascular Aβ accumulation and attenuating Aβ-induced vascular inflammation [16, 17]. Finally, previous work genetically manipulating apoA-I levels in APP/PS1 mice found changes specifically in vascular amyloid deposition without any changes to parenchymal plaques suggesting that the cerebrovasculature is the most sensitive target of HDL in the brain [34, 37]. As described above, we observed several vascular-specific changes in APP/PS1 mice based on apoA-I genotype. Briefly, we found increased protein levels of PDGFRβ and ICAM-1 as well as ICAM-1-positive area on endothelial cells and GFAP-positive astrocytes associated with the cerebrovasculature. That we observed the most prominent effects of apoA-I in the cortex is also consistent the anatomical location of CAA-susceptible penetrating arteries.

Our observations of increased plaque-associated GFAP in apoA-I^KO^ APP/PS1 mice suggests an interaction among Aβ, astrogliosis, and apoA-I. CAA-associated GFAP, but not vascular GFAP-associated CAA, was significantly elevated in apoA-I^KO^ mice. Therefore, we speculate that the loss of apoA-I increases the reactivity of astrocytes to amyloid, as opposed to loss of apoA-I increasing astrogliosis first and then leading to increased amyloid deposition as a result. In other words, the anti-inflammatory effects of HDL in the brain are working specifically against Aβ. In support of this hypothesis, loss of apoA-I did not significantly affect any neuroinflammatory marker in WT mice, which do not accumulate Aβ. Others have similarly observed that the elimination of apoA-I is insufficient to observe pathological vascular changes. For example, apoA-I deficiency alone does not cause atherosclerosis in mice even at 15 months of age on an atherosclerotic diet, whereas apoA-I deficiency can worsen the atherosclerotic pathology of mice lacking LDLR [59]. Interestingly, loss of apoA-I expression may indeed be sufficient to result in hypothalamic astrogliosis [60]; however, we did not observe any significant genotype effects on GFAP expression in the hypothalamus in the current study (Additional file 1: Figure S6a and b).

If the primary mechanism of action of HDL on AD-relevant pathologies is through HDL’s peripheral activities, increasing circulating HDL may be an attractive therapeutic approach that may complement anti-amyloid approaches. Although HDL-based therapeutics have not yet been studied in humans for their potential benefits against cognitive decline, several studies have investigated whether treating vascular risk factors can affect dementia and AD risk. Indeed, a recent systematic review and meta-analysis found that using statins to improve lipid health significantly reduced AD and dementia risk overall in prospective trials; however, the single RCT performed did not find any benefits [61].

Future research on HDL-based therapeutic approaches for AD will benefit from the considerable safety and efficacy data gathered from clinical trials using HDL formulations, including recombinant apoA-I proteins, apoA-I mimetics, and plasma-derived apoA-I, all of which were developed to treat atherosclerosis [62, 63]. CER-001 and MDCO-216 are recombinant proteins of the wildtype and Milano sequences of apoA-I, respectively, which were both well tolerated as infusions in phase II clinical trials but were unsuccessful in meeting the primary endpoint of reduced atherosclerosis [64–66]. ApoA-I mimetic peptides, such as D-4F and L-4F, were developed to overcome the production difficulties associated with full-length recombinant proteins and improve oral bioavailability. Phase I and II clinical trials show that D-4F and L-4F are also well tolerated but ineffective in reducing atherosclerosis [67, 68]. A more promising therapeutic is CSL-112, a plasma-derived, reconstituted apoA-I formulation that was well tolerated in phase I and II clinical trials and showed early indications of improvements to cholesterol efflux capacity [69]. Plasma-derived pre-β HDL has also been tested in an autologous manner whereby delipidated HDL is infused back into the patient from which it was obtained, an approach that is well tolerated and tends to reduce atherosclerosis [70]. Subjects are currently being recruited for phase III trials for both of these plasma-derived HDL formulations. Direct upregulation of apoA-I transcription has also been attempted with the small molecule RVX-208; however, establishing an appropriate dose to balance efficacy and safety has proven difficult [71, 72]. Despite the mixed data on efficacy in treating atherosclerosis, the abundant information on safety and tolerability in clinical trials is promising for the potential repurposing of HDL-based therapeutics for AD.

In addition to the potential of HDL as an AD therapeutic, HDL may be a valuable tool to prevent or treat the cerebrovascular adverse effects of other AD therapies. Anti-Aβ monoclonal antibodies are one of the major drug types currently in phase II and III clinical trials for AD [73]. The success of these drugs is still to be determined through ongoing trials, but it has become clear that amyloid-related imaging abnormalities (ARIA) [74, 75] found on MRI indicating cerebrovascular edema are a potential adverse effect. ARIA in anti-Aβ monoclonal antibody trials is thought to result from the vascular accumulation of Aβ42 that is solubilized from plaques by the therapeutic antibodies and cleared from the brain via periarterial pathways [76]. The present work showing that apoA-I reduces vascular Aβ deposition and the recent work by our group with 3D engineered human arteries showing that HDL can specifically prevent Aβ42 vascular accumulation suggests that HDL may be a valuable companion therapeutic to administer in conjunction with anti-amyloid therapies to prevent or treat ARIA. Our findings also suggest that HDL may be a valuable tool in the treatment of CAA-related inflammation (CAARI). CAARI is a form of CAA presenting as an MRI abnormality similar to ARIA but with an acute onset and the presence of inflammation and amyloid deposition together [77]. We showed in the present study that lack of apoA-I exacerbates astrocyte reactivity to vascular Aβ; therefore, HDL-based therapeutics may have a unique ability to protect against CAARI by suppressing the reactivity of immune cells to CAA.

Our study has several limitations. First, our breeding strategy was designed to maximize the production of mice with a total absence of apoA-I rather than produce control animals with wildtype levels of apoA-I. Nevertheless, our observation of a robust reduction in HDL-C levels between apoA-I^HEM^ and apoA-I^KO^ mice is consistent with previous literature showing that the difference in plasma HDL-C levels is indeed larger in magnitude between apoA-I^HEM^ and apoA-I^KO^ mice compared to apoA-I^HEM^ and apoA-I^WT^ mice. Therefore, we posit that the apoA-I^HEM^ and apoA-I^KO^ cohorts in this study are sufficient to test the hypothesis that reducing plasma HDL-C levels worsens cerebrovascular phenotypes in APP/PS1 mice. Further limitations include use of mixed sexes and analysis at a single age. It is well known that in humans, females have a higher risk of AD than males and their disease progresses more quickly upon diagnosis [78]. Although we were unable to make statistical comparisons based on sex due to limitations in breeding and survival of animals of each genotype and sex, male and female mice are distinguished visually in all of our presented data and no clear sex bias is evident even though the sex of the mice was not balanced between groups. Aging is another major risk factor for AD that we did not extensively investigate, as our study examines mice at 12 months of age when amyloid pathology is well developed. A technical limitation in this study is our limited investigation of the interaction of the vasculature, amyloid, ICAM-1 and astrogliosis by the method of immunofluorescence. While we were conservative in our investigation by only analyzing amyloid, CD31, ICAM-1 and GFAP that were completely associated, it is possible that some association could arise from the overlap of these markers in different planes due to the z-stack imaging technique used to visualize the brain sections. A second technical limitation is that biochemical analysis was only evaluated in crude half-brain homogenates; therefore, our ability to detect region-specific changes was limited for biochemical assessments. Finally, our ability to detect significant genotype effects on memory performance is limited by relatively small cohorts that survived until analysis.

Despite these limitations, this study makes significant progress in developing a strong rationale to test HDL as a therapeutic agent in AD that specifically targets the vasculature. The work by both Lewis et al. and Lefterov et al. showing that genetic manipulation of apoA-I can specifically affect CAA has not been confirmed since the original publications in 2010. Our study also expands upon the role of apoA-I in cerebrovascular health by showing that loss of apoA-I increases Aβ within the brain and exacerbates the potentially pathological reactivity of astrocytes to vascular and parenchymal Aβ. Some groups have already begun to evaluate the potential benefits of apoA-I mimetics or reconstituted HDL in AD mice and have observed benefits with respect to amyloid pathology, CAA, and whole brain neuroinflammation [38–41], although not all studies found significant improvements or reported data for all of these pathologies. Our current study demonstrates that the investigation of vascular-specific pathologies, including CAA and cerebrovascular astrogliosis, may be critical in future studies using HDL-based therapeutics to target AD pathology in general and vascular-specific amyloid pathologies including CAA, ARIA, and CAARI.

## Conclusions

We confirm previous findings that HDL reduces CAA and neuroinflammation and extend previous work by showing that HDL specifically prevents cerebrovascular astrogliosis and astrocyte reactivity to Aβ deposited in both parenchymal plaques and in the cerebrovasculature. This work furthers our understanding of how HDL affects brain health, likely through the circulation, and suggests that targeting systemic HDL may improve cerebrovascular health in humans to protect against amyloid and neuroinflammatory pathologies in AD.

## Additional files

Additional file 1: **Figure S1.** Survival rates for (a) female and (b) male mice with known genotype upon death or experiment endpoint. Log-rank test *p* values are shown below graphs. **Figure S2.** Analysis strategy for histology. Images with bold, colored outlines indicate steps measuring percent positive area. Colored arrows between images indicate steps where the pixels positive in both images were combined into a new image. **Figure S3.** Morphological discrimination of vascular versus parenchymal amyloid for analysis of CAA area. Examples of inverted 8-bit X-34 images are shown with (right) and without (left) vascular (teal boxes) and parenchymal (green circles) amyloid indicated. **Figure S4.** Total vascular area was unchanged by APP/PS1 or apoA-I genotype. Vessel area (a, c), thickness (e, f) and tortuosity (g, h) as defined by immunofluorescent staining of CD31 in the (a, e, g) cortex and (c, f, h) hippocampus. (b, d) Representative images. **Figure S5.** Contextual and cued fear memory was not significantly affected by APP/PS1 or apoA-I genotype. Mice were trained to associate a context or auditory cue with a foot shock then tested for memory of the associated (a) context or (b) cue as measured by the time spent freezing Omnibus analyses of apoA-I and APP/PS1 genotype effects by two-way ANOVA are displayed as exact *p* values below graphs. **Figure S6.** Hypothalamic GFAP staining area was unaffected by apoA-I and APP/PS1 genotype. (a) GFAP staining area was visualized by immunofluorescence in the hypothalamus. (b) Representative image. For graphs in Figure S4, S5, and S6, points represent individual mice and bars represent mean values, circles represent female mice, squares represent male mice, and *N* = 5-23 mice per genotype. apoA-I, apolipoprotein A-I; HEM, hemizygous apoA-I genotype; KO, knockout apoA-I genotype; WT, wildtype APP/PS1 genotype; APP/PS1, transgenic APP/PS1 genotype; CD31, cluster of differentiation 21; GFAP, glial fibrillary acidic protein. (PDF 2870 kb)

Additional file 2: Macro text for the quantification of total amyloid, GFAP, and vascular area, vascular astrogliosis, GFAP-associated plaques, CAA, and CAA-associated GFAP. (DOCX 22 kb)

## Abbreviations

ABCA1: Adenosine triphosphate binding cassette transporter A1
AD: Alzheimer’s disease
apoA-I: Apolipoprotein A-I
Aβ: Amyloid beta
CAA: Cerebral amyloid angiopathy
CD31: Cluster of differentiation 31
GFAP: Glial fibrillary acidic protein
HDL: High-density lipoproteins
HDL-C: High-density lipoprotein cholesterol
ICAM-1: Intercellular adhesion molecule 1
IL-1β: Interleukin 1 beta
IL-6: Interleukin 6
LDL-C: Low-density lipoprotein cholesterol
PDGFRβ: Platelet-derived growth factor receptor beta
TNFα: Tumor necrosis factor alpha
VCAM-1: Vascular cell adhesion molecule 1
