# Supplementary material for: ApoA-I deficiency increases cortical amyloid deposition, cerebral amyloid angiopathy, cortical and hippocampal astrogliosis, and amyloid-associated astrocyte reactivity in APP/PS1 mice
Source: Alzheimers Res Ther. 2019 May 13;11:44. doi: 10.1186/s13195-019-0497-9 (PMC6515644; doi:10.1186/s13195-019-0497-9)

Supplemental Figure 1

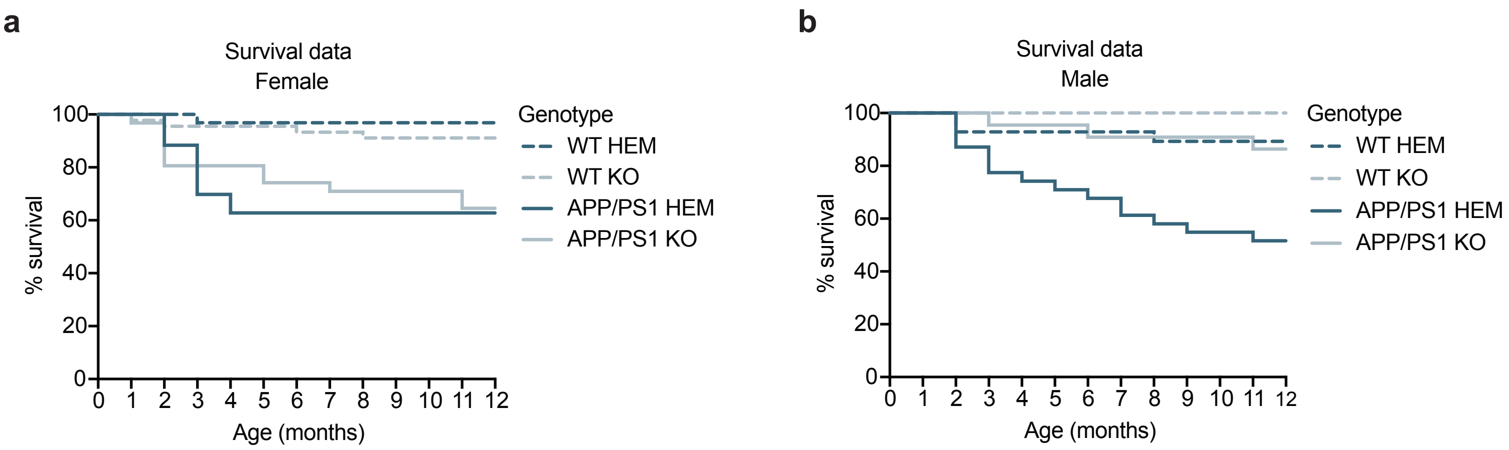

Log-rank test, (Bonferroni correction)
APP/PS1 KO female vs APP/PS1 KO male: p=0.031, (p=0.372)
APP/PS1 HEM female vs WT HEM female: p=0.001, (p=0.008)
APP/PS1 KO female vs WT KO female: p=0.013, (p=0.151)
APP/PS1 HEM male vs WT HEM male: p=0.002, (p=0.019)
APP/PS1 HEM male vs APP/PS1 KO male: p=0.005, (p=0.055)

Supplemental Figure 2

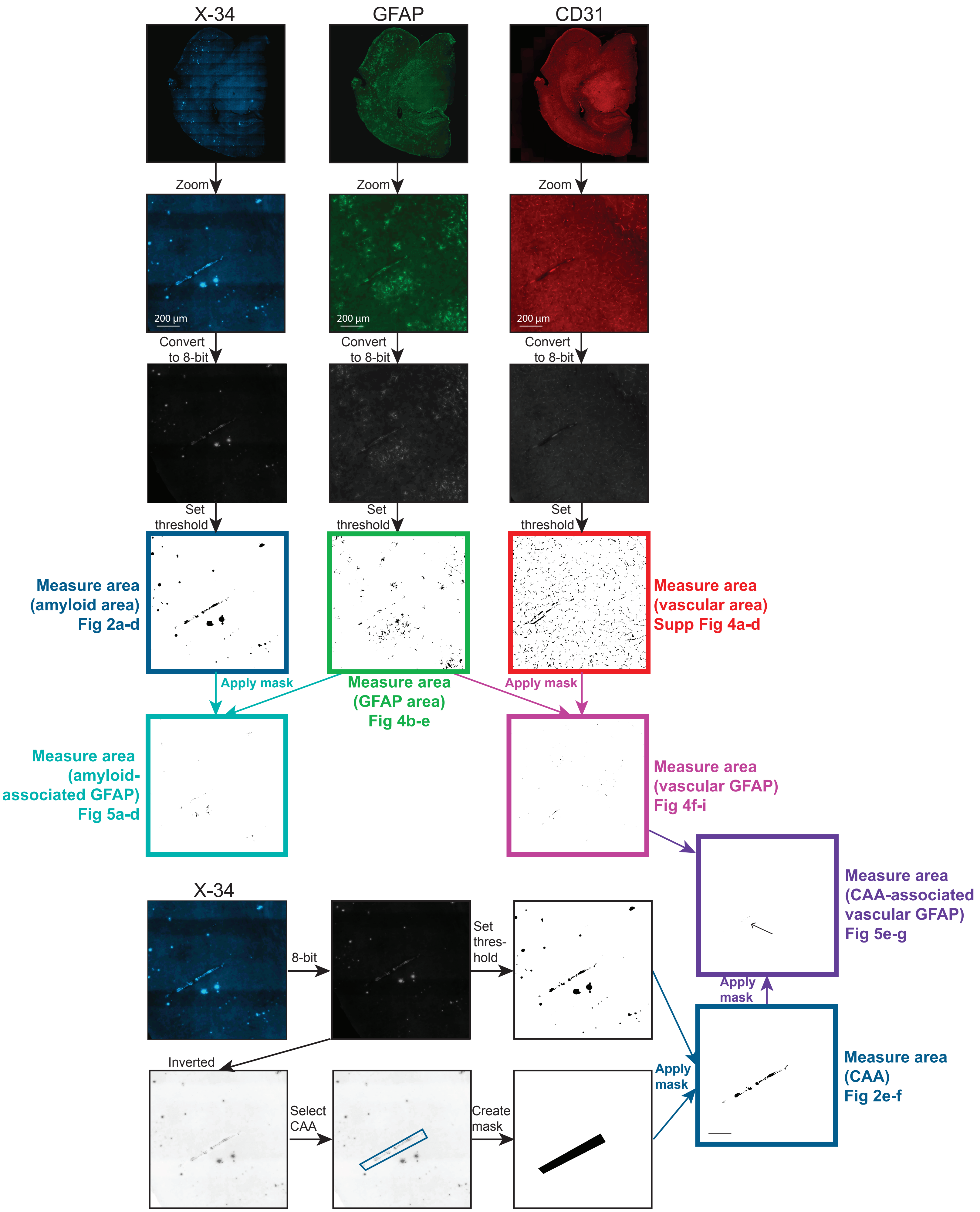

Supplemental Figure 3

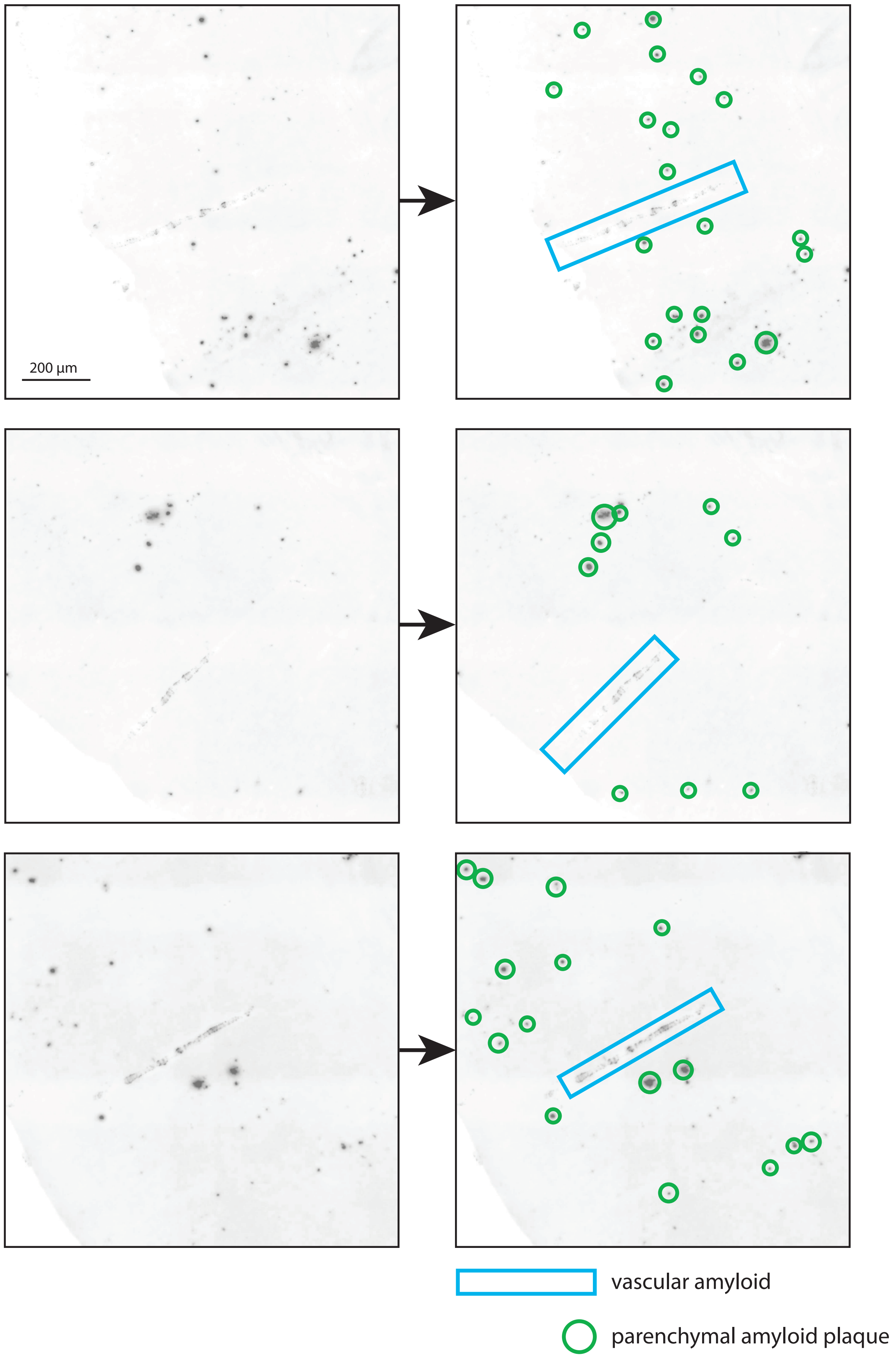

Supplemental Figure 4

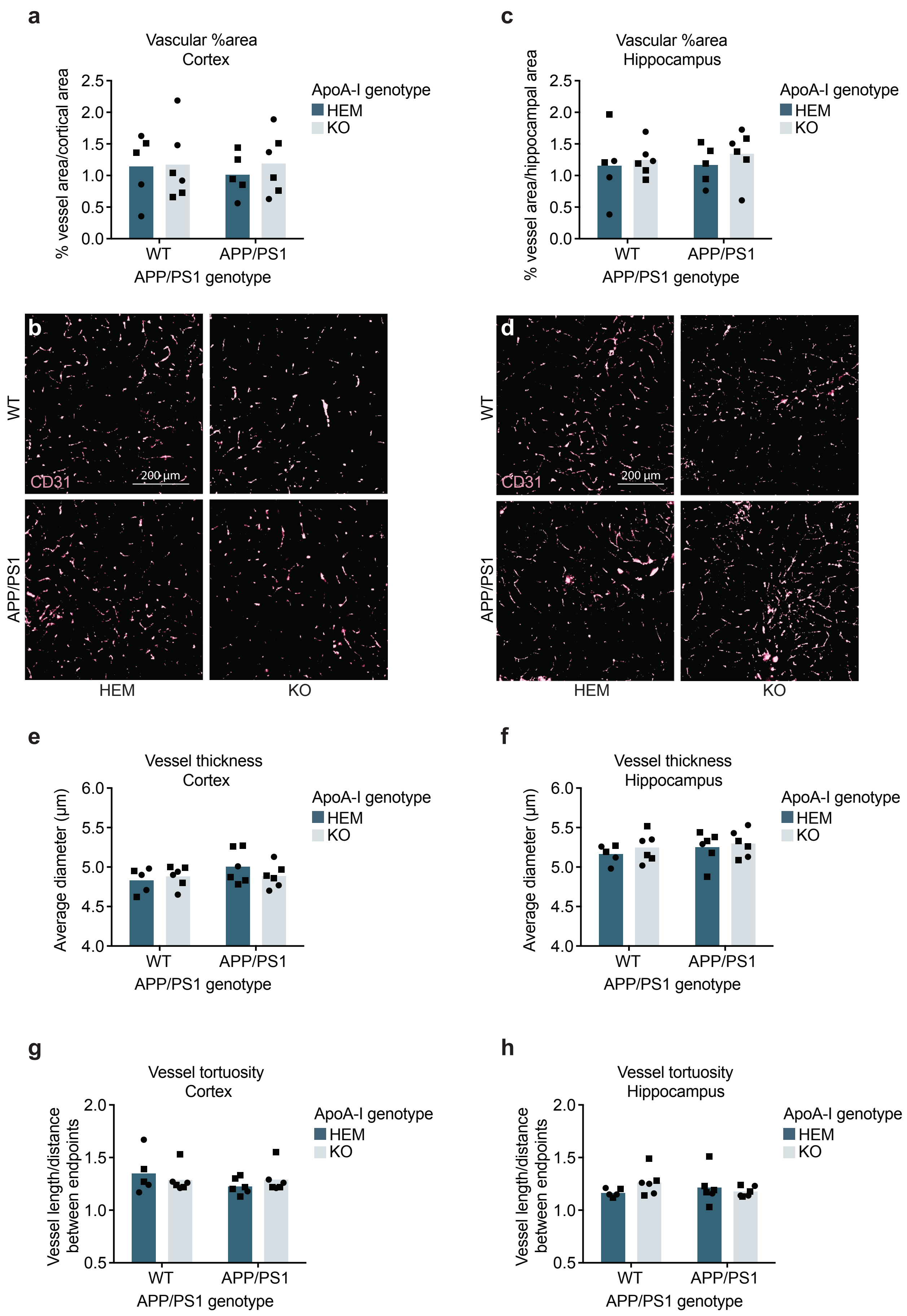

Supplemental Figure 5

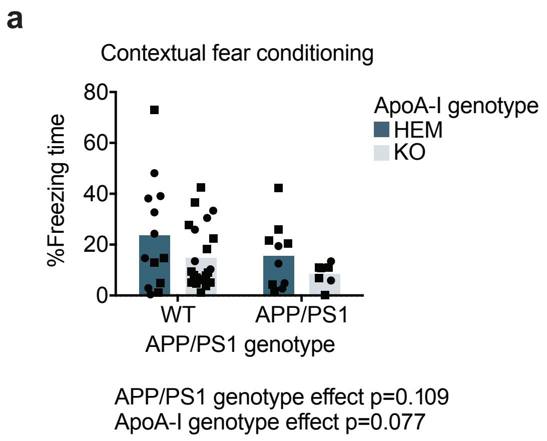

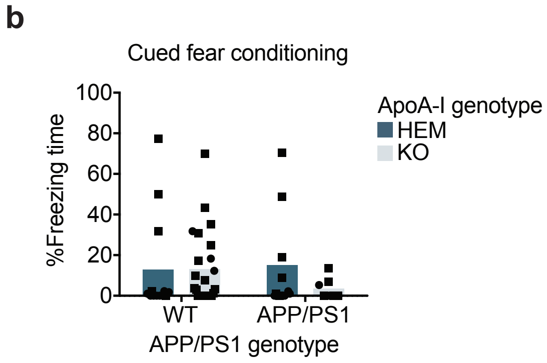

Supplemental Figure 6

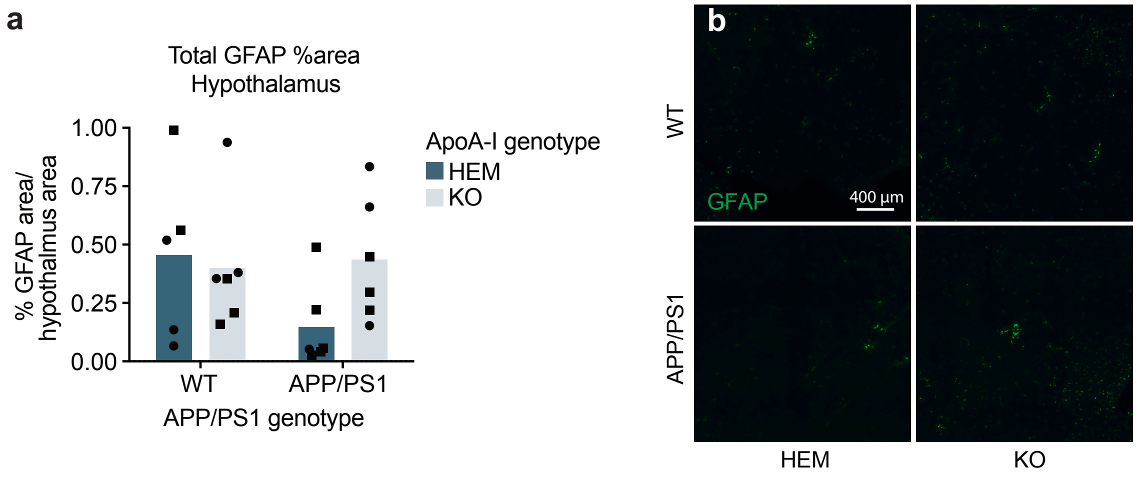

Supplement: Supplementary file 1 — Figure S1. Survival rates for (a) female and (b) male mice with known genotype upon death or experiment endpoint. Log-rank test p values are shown below graphs. Figure S2. Analysis strategy for histology. Images with bold, colored outlines indicate steps measuring percent positive area. Colored arrows between images indicate steps where the pixels positive in both images were combined into a new image. Figure S3. Morphological discrimination of vascular versus parenchymal amyloid for analysis of CAA area. Examples of inverted 8-bit X-34 images are shown with (right) and without (left) vascular (teal boxes) and parenchymal (green circles) amyloid indicated. Figure S4. Total vascular area was unchanged by APP/PS1 or apoA-I genotype. Vessel area (a, c), thickness (e, f) and tortuosity (g, h) as defined by immunofluorescent staining of CD31 in the (a, e, g) cortex and (c, f, h) hippocampus. (b, d) Representative images. Figure S5. Contextual and cued fear memory was not significantly affected by APP/PS1 or apoA-I genotype. Mice were trained to associate a context or auditory cue with a foot shock then tested for memory of the associated (a) context or (b) cue as measured by the time spent freezing Omnibus analyses of apoA-I and APP/PS1 genotype effects by two-way ANOVA are displayed as exact p values below graphs. Figure S6. Hypothalamic GFAP staining area was unaffected by apoA-I and APP/PS1 genotype. (a) GFAP staining area was visualized by immunofluorescence in the hypothalamus. (b) Representative image. For graphs in Figure S4, S5, and S6, points represent individual mice and bars represent mean values, circles represent female mice, squares represent male mice, and N = 5-23 mice per genotype. apoA-I, apolipoprotein A-I; HEM, hemizygous apoA-I genotype; KO, knockout apoA-I genotype; WT, wildtype APP/PS1 genotype; APP/PS1, transgenic APP/PS1 genotype; CD31, cluster of differentiation 21; GFAP, glial fibrillary acidic protein. (PDF 2870 kb) [file 13195_2019_497_MOESM1_ESM.pdf]
