# Supplementary material for: ApoA-I deficiency increases cortical amyloid deposition, cerebral amyloid angiopathy, cortical and hippocampal astrogliosis, and amyloid-associated astrocyte reactivity in APP/PS1 mice
Source: Alzheimers Res Ther. 2019 May 13;11:44. doi: 10.1186/s13195-019-0497-9 (PMC6515644; doi:10.1186/s13195-019-0497-9)
Supplement: Supplementary file 2 — Macro text for the quantification of total amyloid, GFAP, and vascular area, vascular astrogliosis, GFAP-associated plaques, CAA, and CAA-associated GFAP. (DOCX 22 kb) [file 13195_2019_497_MOESM2_ESM.docx]

**Macros for immunofluorescence analysis**

Below is the text to be used in the ImageJ macro system (IJ1 macro language) for the automated analysis of all area and markers described in the manuscript. Before running macros, regions of interest must manually be defined, saved as an ROI set, and the ROI set must be open. Image files must also be organized in the image folder as described in each macro. Minimum and maximum thresholds for each channel must also be determined manually then inserted into the macro where indicated.

***Macro #1: total cortical and hippocampal GFAP, X-34, and CD31 area; vessel-association GFAP in the cortex and hippocampus; X-34 plaque-associated GFAP in the cortex and hippocampus***

| //images are organized in the following order for each brain section: GFAP, CD31, merged, X-34 |
| --- |
|  |
| input="*(insert mapping to image folder here)*"; |
|  |
| images = getFileList(input); |
| images = Array.sort(images); |
|  |
| GFAPmin = *(insert threshold min for GFAP here)* |
| GFAPmax = *(insert threshold max for GFAP here)* |
| X34min = *(insert threshold min for X-34 here)* |
| X34max = *(insert threshold max for X-34 here)* |
|  |
| //i=CD31 image |
| //j=GFAP image |
| //k=ROI hippocampus, ROIset already open and organized (list order cortex then hippocampus for each sections) |
| //l=ROI cortex, ROIset already open and organized (list order cortex then hippocampus for each sections) |
| //m=X-34 image |
|  |
| for (i=3; i<images.length; i+=4) { |
| j=i-3; |
| m=i-1; |
|  |
| if (j==0){ |
| l=0; |
| k=1; |
| } else { |
| l=j/2; |
| k=l+1; |
| } |
|  |
| //make mask of CD31 |
| open(input+images[i]); |
| run("8-bit"); |
| run("Auto Local Threshold", "method=Bernsen radius=15 parameter_1=0 parameter_2=0 white"); |
| run("Create Mask"); |
| close(images[i]); |
|  |
| //measure GFAP within CD31 mask |
|  |
| //convert GFAP into mask |
| open(input+images[j]); |
| print("opening image "+images[j]); |
| run("8-bit"); |
| setThreshold(GFAPmin, GFAPmax); |
| run("Convert to Mask"); |
| imageCalculator("AND create", images[j],"mask"); |
|  |
| //measure GFAP within CD31 mask, cortex then hippocampus |
| selectWindow("Result of "+images[j]); |
| roiManager("Select", l); |
| run("Analyze Particles...", "clear summarize"); |
| roiManager("Select", k); |
| run("Analyze Particles...", "clear summarize"); |
|  |
| //measure CD31 mask area within ROI, cortex then hippocampus |
| selectWindow("mask"); |
| roiManager("Select", l); |
| run("Analyze Particles...", "clear summarize"); |
| roiManager("Select", k); |
| run("Analyze Particles...", "clear summarize"); |
|  |
| //measure GFAP area within cortical or hippocampal ROI, cortex then hippocampus |
| selectWindow(images[j]); |
| roiManager("Select", l); |
| run("Analyze Particles...", "clear summarize"); |
| roiManager("Select", k); |
| run("Analyze Particles...", "clear summarize"); |
|  |
| close(images[j]); |
| close("Result of "+images[j]); |
|  |
| //measure X-34 area within ROI |
| open(input+images[m]); |
| print("opening image "+images[m]); |
| run("8-bit"); |
| setThreshold(X34min, X34max); |
| selectWindow(images[m]); |
| roiManager("Select", l); |
| run("Analyze Particles...", "clear summarize"); |
| roiManager("Select", k); |
| run("Analyze Particles...", "clear summarize"); |
|  |
| //measure GFAP around plaques |
|  |
| //make mask of X-34 |
| selectWindow(images[m]); |
| run("Create Mask"); |
|  |
| //convert GFAP into X-34 mask |
| open(input+images[j]); |
| print("opening image "+images[j]); |
| run("8-bit"); |
| setThreshold(GFAPmin, GFAPmax); |
| run("Convert to Mask"); |
| imageCalculator("AND create", images[j], "mask"); |
|  |
| //measure GFAP in X-34 mask, cortex then hippocampus |
| roiManager("Select", l); |
| run("Analyze Particles...", "clear summarize"); |
| roiManager("Select", k); |
| run("Analyze Particles...", "clear summarize"); |
|  |
|  |
| run("Close All"); |
|  |
| } |

***Macro #2: cerebral amyloid angiopathy (CAA) area and GFAP-associated CAA***

| //images are organized in the following order for each brain sections: GFAP, CD31, merged, X-34 |
| --- |
|  |
| input="*(insert mapping to image folder here)*"; |
|  |
| images = getFileList(input); |
| images = Array.sort(images); |
|  |
| GFAPmin = *(insert threshold min for GFAP here)* |
| GFAPmax = *(insert threshold max for GFAP here)* |
| X34min = *(insert threshold min for X-34 here)* |
| X34max = *(insert threshold max for X-34 here)* |
|  |
|  |
| for (i=3; i<images.length; i++) { |
| print(images[i]); |
| } |
|  |
| CAAinput="(insert mapping to CAA manual mask folder here)"; |
| CAAmask = getFileList(CAAinput); |
| CAAmask = Array.sort(CAAmask); |
| for (k=0; k<CAAmask.length; k++) { |
| print(images[k]); |
| } |
|  |
| //i=CD31 image |
| //j=GFAP image |
| //m=X-34 image |
| //k=CAA mask |
| //l=ROIcortex |
|  |
| for (i=3; i<images.length; i+=4) { |
| j=i-3; |
| m=i-1; |
|  |
| if (j==0) { |
| k=0; |
| l=0; |
| } else { |
| k=(j/4); |
| l=j/4; |
| } |
|  |
| //open mask |
| open(CAAinput+CAAmask[k]); |
|  |
| //convert X-34 into CAA manual mask |
| open(input+images[m]); |
| print("opening image "+images[m]); |
| run("8-bit"); |
| setThreshold(X34min, X34max); |
| run("Convert to Mask"); |
| imageCalculator("AND create", images[m],CAAmask[k]); |
| setThreshold(1, 255); |
| run("Create Mask"); |
|  |
| //measure X-34 in CAA manual mask |
| roiManager("Select", l); |
| run("Analyze Particles...", "clear summarize"); |
|  |
| close(images[m]); |
| close("Result of "+images[m]); |
| close(CAAmask[k]); |
|  |
| //create GFAP in CD31 mask |
| //open CD31 |
| open(input+images[i]); |
| run("8-bit"); |
| run("Auto Local Threshold", "method=Bernsen radius=15 parameter_1=0 parameter_2=0 white"); |
|  |
| //convert GFAP into CD31 |
| open(input+images[j]); |
| print("opening image "+images[j]); |
| run("8-bit"); |
| setThreshold(GFAPmin, GFAPmax); |
| run("Convert to Mask"); |
| imageCalculator("AND create", images[j], images[i]); |
|  |
| //convert CAA into GFAP vascular mask |
| run("Convert to Mask"); |
| imageCalculator("AND create", "mask", "Result of "+images[j]); |
|  |
| //measure vascular GFAP and CAA co-localization |
| roiManager("Select", l); |
| run("Analyze Particles...", "clear summarize"); |
|  |
|  |
| run("Close All"); |
|  |
|  |
| } |

***Macro #3: total GFAP area in the hypothalamus***

| //images are organized in the following order for each brain sections: GFAP, merged, CD31 |
| --- |
|  |
| input="*(insert mapping to image folder here)*"; |
|  |
| images = getFileList(input); |
| images = Array.sort(images); |
|  |
| GFAPmin = *(insert threshold min for GFAP here)* |
| GFAPmax = *(insert threshold max for GFAP here)* |
| //j=GFAP image |
| //k=ROI hypothalamus, ROIset already open and organized |
|  |
| for (i=2; i<images.length; i+=3) { |
| j=i-2; |
|  |
| if (j==0){ |
|  |
| k=0; |
| } else { |
| k=j/3; |
| } |
|  |
| //measure GFAP area within cortical or hippocampal ROI, cortex then hippocampus |
| open(input+images[j]); |
| run("8-bit"); |
| setThreshold(GFAPmin, GFAPmax); |
| run("Convert to Mask"); |
| roiManager("Select", k); |
| run("Analyze Particles...", "clear summarize"); |
|  |
| run("Close All"); |
|  |
| } |
